# Multimodal functional deep learning for multiomics data

**DOI:** 10.1093/bib/bbae448

**Published:** 2024-09-16

**Authors:** Yuan Zhou, Pei Geng, Shan Zhang, Feifei Xiao, Guoshuai Cai, Li Chen, For the Alzheimer’s Disease Neuroimaging Initiative, Qing Lu

**Affiliations:** Department of Biostatistics, University of Florida, 2004 Mowry Rd, Gainesville, FL 32611, USA; Department of Mathematics and Statistics, University of New Hampshire, 33 Academic Way, Durham, NH 03824, USA; Department of Statistics and Probability, Michigan State University, 619 Red Cedar Road, East Lansing, MI 48824, USA; Department of Biostatistics, University of Florida, 2004 Mowry Rd, Gainesville, FL 32611, USA; Department of Surgery, University of Florida, Gainesville, 1600 SW Archer Rd, FL 32611, USA; Department of Biostatistics, University of Florida, 2004 Mowry Rd, Gainesville, FL 32611, USA; Department of Biostatistics, University of Florida, 2004 Mowry Rd, Gainesville, FL 32611, USA

**Keywords:** multiomics inputs, deep learning, functional data analysis

## Abstract

With rapidly evolving high-throughput technologies and consistently decreasing costs, collecting multimodal omics data in large-scale studies has become feasible. Although studying multiomics provides a new comprehensive approach in understanding the complex biological mechanisms of human diseases, the high dimensionality of omics data and the complexity of the interactions among various omics levels in contributing to disease phenotypes present tremendous analytical challenges. There is a great need of novel analytical methods to address these challenges and to facilitate multiomics analyses. In this paper, we propose a multimodal functional deep learning (MFDL) method for the analysis of high-dimensional multiomics data. The MFDL method models the complex relationships between multiomics variants and disease phenotypes through the hierarchical structure of deep neural networks and handles high-dimensional omics data using the functional data analysis technique. Furthermore, MFDL leverages the structure of the multimodal model to capture interactions between different types of omics data. Through simulation studies and real-data applications, we demonstrate the advantages of MFDL in terms of prediction accuracy and its robustness to the high dimensionality and noise within the data.

## Introduction

Advances in high-throughput technologies have enabled us to collect enriched multiomics datasets that capture the high-dimensional and complex variations at various omics levels. This collected multimodal data of omics, which includes the genome, epigenome, transcriptome, proteome, metabolome, etc., allows for a systematic study of how different omics levels act jointly to affect human diseases. While the emerging multiomics datasets hold great promise for enhancing our understanding of these diseases, the high dimensionality, complex inter-relationships, low signal-to-noise ratio, and issues with data quality (e.g. missing values) in the multiomics data pose considerable analytical challenges [1].

Over the past two decades, a variety of methods have been developed for multiomics data analysis: methods such as Similarity Network Fusion [2] and mixOmics [3], select, extract, and integrate features of multiomics. Other tools, like MultiOmics Factor Analysis [4] and miodin [5], integrate data based on factor analysis, which could have computational efficiency issues [6]. To alleviate the computational burden, dimension reduction techniques have been widely applied in multiomics data analysis [7]. Commonly used dimension reduction approaches include extensions of classical methods [8], such as penalized canonical correlation analysis (CCA) [9], sparse CCA [10], generalized SVD [11], co-inertia analysis (CIA) [12], sparse extensions of partial least squares (PLS) [13], and the self-paced learning L1/2 absolute network-based logistic regression model (SLNL) [14]. However, these approaches have inherent limitations on sparse data. More recently, the state-of-the-art machine learning (ML) methods have been increasingly used in multiomics data, including a novel MultiOmics Meta-learning Algorithm (MUMA) [15] and other methods reviewed by Chung et al. [16]. Although ML-based feature selection approaches [17] and ML-based clustering methods [18] have notably tackled the computational efficiency challenges of high-dimensional datasets; most ML methods still suffer from overfitting problems when integrating multiomics datasets [6]. In a recent report [19], we developed a new functional neural network (FNN) method that incorporates functional data analysis (FDA) techniques to account for the underlying structure of genetic data, such as the linkage disequilibrium (LD) among neighboring variants, which successfully alleviates the over-fitting issue in high-dimensional genetic data. In this study, we propose a multimodal functional deep learning (MFDL) method to facilitate multiomics data analysis, with the advantages in over-fitting control, genetic structure modeling, and multiomics data integration, which will be illustrated in detail below.

In the proposed MFDL method, we introduce an omics variant function by fitting a series of basis functions to each type of omics data (e.g. genome, epigenome, transcriptome, etc.) in the input layer, then integrate these fitted functions into the dimension-reduced hidden layers as a shared representation. From this shared representation, additional hidden layers are formed to continue the training of the model and to learn the complex relationships between multiomics and the phenotype of interest. The MFDL model has the following unique advantages: (i) it inherits the robustness of the FNN method to high-dimensional datasets and low signal-to-noise ratios by utilizing FDA techniques; (ii) its flexible multimodal structure allows to learn a shared representation through the hidden layers of deep neural networks, which facilitates the capture of interactions and correlations between multiple omics inputs. (iii) The MFDL model can analyze outcomes in various forms (e.g. scalar, vector, or functional outcomes) and complex nonlinear relationships between outcomes and multiomics inputs. Through simulation studies and two real data applications, we demonstrate the superiority of the MFDL models in terms of both accuracy and robustness compared to the functional linear model (FLM), FNN, and feedforward artificial neural networks (NN) in multiomics data analysis.

The paper is organized as follows: section “System and Methods” introduces the MFDL model with a brief overview of the FLM and the FNN method. In section “Simulation studies,” we conduct three simulations to compare the performance of the proposed MFDL with FLM and FNN under various simulation settings. In section “Simulation Settings,” we demonstrate the MFDL through two real data applications. Section “Discussion” discusses the merits of the proposed method and future directions. Technical details are included in Appendix 1.

## System and Methods

To motivate the MFDL model, we first introduce the FLM and FNN methods for genetic data analysis along with the notation used in this paper. Building on these methods, we propose the MFDL model to accommodate multiple omics inputs and complex phenotypes.

For the $i$-th individual of the study, we denote ${y}_i$ as phenotype and ${g}_{ki}=\left({g}_{ki1},{g}_{ki2},\cdots, {g}_{ki{p}_k}\right)$ as the $k$-th omics input with dimension ${p}_k$ for $i=1,\dots, n$ and $k=1,\dots, m$. Without loss of generality, for the rest of the paper, we state the models in the case of $m=2.$

### Functional linear model

A functional linear model (FLM) can be constructed from a traditional linear model by substituting the vector of covariate observations with functional covariates, provided that at least one of the following conditions holds: (i) the dependent or response variable is considered functional and (ii) one or more of the independent variables or covariates are considered functional [20]. In the context of genetic data analysis, to evaluate the joint association of multiple omics levels with a disease phenotype, an FLM can be formulated by incorporating multiomics variants as functional covariates [21].

For each type of omics data with available location information (e.g. a gene), we scale location information to [0, 1], denoted as ${t}_k$. We construct the omics variant function ${G}_{ki}\left({t}_k\right)$ using a linear combination of the Dirac Delta function [19]. The FLM incorporating two functional inputs ${G}_{1i}\left({t}_1\right)\kern0.5em \mathrm{and}\ {G}_{2i}\left({t}_2\right)$ can be expressed as


(2.1) \begin{equation*} {y}_i={\alpha}_0+{X}_i\alpha +{\int}_0^1{G}_{1i}\left({t}_1\right){\beta}_1\left({t}_1\right)d{t}_1+{\int}_0^1{G}_{2i}\left({t}_2\right){\beta}_2\left({t}_2\right)d{t}_2+{\epsilon}_i \end{equation*}

where ${\alpha}_0$ is the overall mean, $\alpha$ is the regression coefficient of covariates, and ${\beta}_1\left({t}_1\right)\ \mathrm{and}\ {\beta}_2\left({t}_2\right)$ are the functional genetic effects of ${G}_{1i}\left({t}_1\right)\ \mathrm{and}\ {G}_{2i}\left({t}_2\right)$. ${\epsilon}_i$ is an error term that is normally distributed.

### Functional neural network

**Figure 1:**
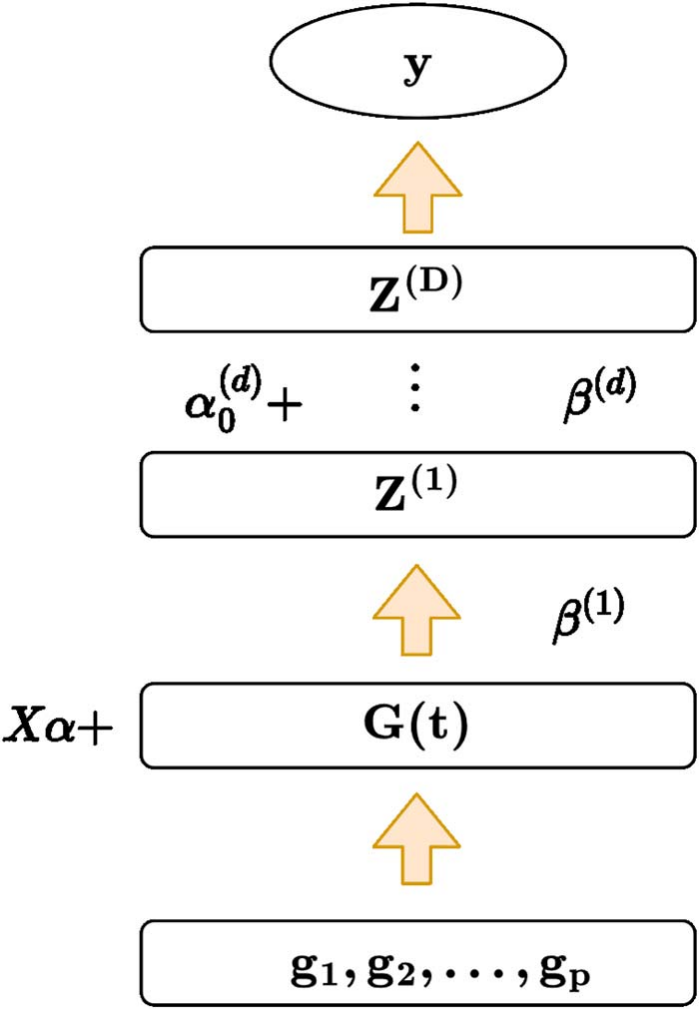
The hierarchical structure of FNN with D hidden layers.

The functional neural network (FNN) model we previously developed was constructed based on the hierarchical structure shown in Fig. 1, where $X$ and $\alpha$ are covariates (e.g. gender) and their corresponding coefficients, respectively. ${\beta}^{(d)}(s)$ and ${\alpha}_0^{(d)}$ refer to the functional weight and scalar bias at $d$-th hidden layer, which can be estimated by backward propagation in functional form. The term ${Z}^{(d)}$refers to the hidden function at the $d$-th hidden layer, which captures nonlinear and nonadditive effects by applying nonlinear activation functions. Technique details can be found in Zhang *et al.* [19].

The FNN model has been proven to offer certain advantages for genetic data analysis including the ability to capture the complex relationships between a single source of genetic variants and disease phenotypes and the flexibility to handle various types of phenotypes. However, when dealing with multiple sources of omics data, the discretized matrices of the omics variant functions must be concatenated as the input in the FNN model. This process may reduce the model’s ability to capture correlations between inputs. To overcome this limitation, we propose the multimodal functional DL model, which features a novel network structure that better accommodates multiple inputs.

### Multimodal functional DL model

**Figure 2:**
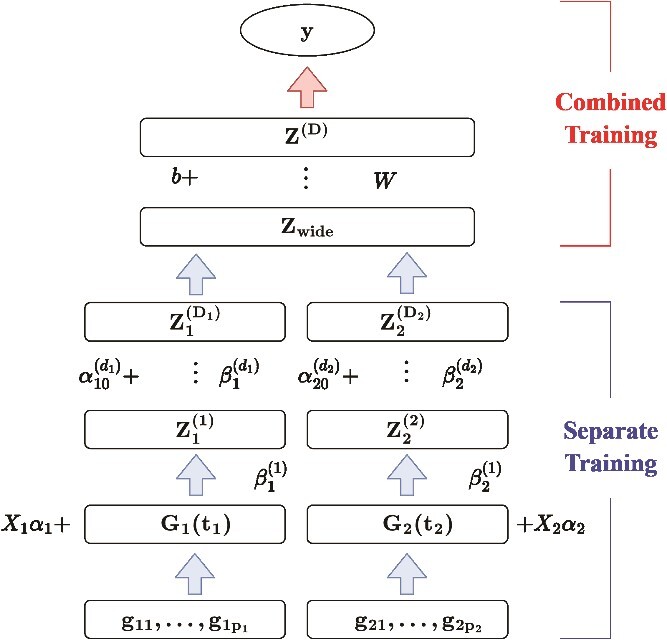
The hierarchical structure of MFDL with two omics input.

Multiomics datasets, collected from diverse biological features (e.g. genetic variation, gene expression, methylation, etc.), exhibit complementary and heterogeneous properties. A multimodal structure can leverage these properties to exploit the correlations between different data sources and improve prediction performance [22]. As shown in Fig. 2, the model we propose consists of two parts. In the separate training part, we train an FNN model for each modality using the functional data analysis technique to account for the LD effect and to reduce data dimensionality. In the combined training part, we further build a shared representation layer that encapsulates complex correlations between omics features, and construct another feedforward neural network model on the shared representation to model the complex relationship between multiomics and the phenotype of interest, considering possible interactions. The structural details of conventional neural networks and FNN can be found in [23] and [19], respectively.

Specifically, we construct the omics variant function and omics effect function denoted as ${G}_{ki}\left({t}_k\right)$ and ${\beta}_{ki}\left({t}_k\right),k=1,2$. We then use the discretized forms of these functions as inputs to form separate FNN models with ${D}_k$ hidden layers ${Z}_{ki}^{(1)},\dots, {Z}_{ki}^{\left({D}_k\right)}$ as follows:


$$ {Z}_{ki}^{(1)}\left({t}_k^{(1)}\right)={\sigma}_k\left({X}_{ki}{\alpha}_k+\int{G}_{ki}\left({t}_k\right){\beta}_k^{(1)}\left({t}_k^{(1)},{t}_k\right)d{t}_k\right), $$

\begin{align*} {Z}_{ki}^{\left({d}_k\right)}\left({t}_k^{\left({d}_k\right)}\right)=&\sigma \left({\alpha}_{k0}^{\left({d}_k\right)}\left({t}_k^{\left({d}_k\right)}\right)+\int{Z}_{ki}^{\left({d}_k-1\right)}(s){\beta}_k^{\left({d}_k\right)}\left({t}_k^{\left({d}_k\right)},{t}_k^{\left({d}_k-1\right)}\right)\right.\\[8pt]& d\ \left.{t}_k^{\left({d}_k-1\right)}\right),\kern0.5em {d}_k=2,\dots, {D}_k-1, \end{align*}

$$ {Z}_{ki}^{\left({D}_k\right)}=f\left({\alpha}_{k0}^{\left({D}_k\right)}+\int{Z}_{ki}^{\left({D}_k-1\right)}\left({t}_k^{\left({D}_k-1\right)}\right){\beta}_k^{\left({D}_k\right)}\left({t}_k^{\left({D}_k-1\right)}\right)d\ {t}_k^{\left({D}_k-1\right)}\right), $$

where ${X}_{ki}$ and ${\alpha}_k$ represent the covariates and their corresponding coefficients, respectively. The terms ${\alpha}_{k0}^{\left({d}_k\right)}(t),\kern0.5em {\beta}_k^{\left({d}_k\right)}\left(s,t\right)$denote the functional bias and weights at the ${d}_k$-th hidden layer of the FNN model, respectively. The function $\sigma$ represents the activation function for the hidden layer, while the $f$ function on the output layer is the linear link function. The explicit form of the bias and weight functions can be expressed as,


$$ {\alpha}_{k0}^{\left({d}_k\right)}\left({t}_k^{\left({d}_k\right)}\right)=\sum_{j=1}^{J^{\left({d}_k\right)}}{b}_{kj}^{\left({d}_k\right)}{\eta}_j^{\left({d}_k\right)}\left({t}_k^{\left({d}_k\right)}\right),\qquad\qquad\qquad $$

$$ {\beta}_k^{\left({d}_k\right)}\left({t}_k^{\left({d}_k\right)},{t}_k^{\left({d}_k-1\right)}\right)=\sum_{j=1}^{J^{\left({d}_k\right)}}\sum_{\ell =1}^{J^{\left({d}_k-1\right)}}{w}_{k\ell j}^{\left({d}_k\right)}{\eta}_{\ell}^{\left({d}_k-1\right)}\left({t}_k^{\left({d}_k-1\right)}\right){\eta}_j^{\left({d}_k\right)}\left({t}_k^{\left({d}_k\right)}\right) $$

where ${w}_k^{\left({d}_k\right)}$ and ${b}_k^{\left({d}_k\right)}$ are parameters that need to be estimated. ${\eta}_j$’s are predetermined basis functions, and ${J}^{\left({d}_k\right)}$ is the number of basis functions at the ${d}_k$-th hidden layer.

After the forward propagation process through all the hidden layers in each FNN model, we concatenate the outputs of the last hidden layer, ${Z}_{ki}^{\left({D}_k\right)},k=1,2$ to form a shared representation ${Z}_{wide,i}$.


$$ {Z_i}^{(1)}={Z}_{wide,i}=\left[{Z}_{1i}^{\left({D}_1\right)}\kern0.5em {Z}_{2i}^{\left({D}_2\right)}\right] $$

Finally, a feedforward NN with $D$ hidden layers is trained on the ${Z}_{wide,i}$ to obtain the fitted phenotype $\hat{y}$.


$$ {Z_i}^{(d)}\!=\sigma \left({b}^{(d)}+{Z}_i^{\left(d-1\right)}{w}^{(d)}\right),d=2,\dots, D-1, {\hat{y}}_i=f\left({b}^{(D)}+{Z_i}^{\left(D-1\right)}{w}^{(D)}\right)\!, $$

where ${w}^{(d)}$and ${b}^{(d)}$ represent the vector weights and scalar bias at the $d$-th hidden layer, respectively.

It is worth mentioning that the proposed MFDL framework is flexible to fit various types of inputs depending on the nature of the omics data. For instance, if the input is a scalar (e.g. gene expression of a single gene) or multivariate with low dimensions (e.g. gene expression data of a limited number of genes), which are not suitable for functional smoothing, those functional weights, and biases ${\beta}_k^{\left({d}_k\right)}\left({t}_k^{\left({d}_k\right)},{t}_k^{\left({d}_k-1\right)}\right)$ and ${\alpha}_{k0}^{\left({d}_k\right)}\left({t}_k^{\left({d}_k\right)}\right)$ in MFDL can be reduced to matrices and vectors ${w}^{\left({d}_k\right)}$and ${b}^{\left({d}_k\right)}$ using linear basis. Under such a circumstance, the MFDL is degenerated to a deep neural network.

The training process of the model is described in Algorithm 1. Specifically, to train the MFDL and estimate the model parameters defined in the previous forward propagation process, we denote the parameters of interest as


$$ W=\left[{w}_k^{\left({d}_k\right)}\!,{w}^{(d)}\right]\!,\ b=\left[{b}_k^{\left({d}_k\right)}\!,{b}^{(d)}\!\right]\!,\kern0.2em k=1,2;{d}_k=1,\dots, {D}_k;d=1,\dots, D $$

**Algorithm 1:** Training process of MFDL for two omics inputs.

Input: $\boldsymbol{{g}_1}$, $\boldsymbol{{t}_1}$, $\boldsymbol{{g}_2}$, $\boldsymbol{{t}_2}$, ${X}_1$, ${X}_2$, y
Output: $\hat{y}$, W, b
Initialization: Construct genetic variant functions ${G}_1$ and ${G}_2$
**while** the objective function is not converged **do**
1: Construct functional neural network (FNN) for ${G}_1$ and ${G}_2$ separately;
2: Feed forward both FNN models to obtain intermediate output ${Z}_1^{\left({D}_1\right)}= FN{N}_1\left({G}_1\right)$, ${Z}_2^{\left({D}_2\right)}= FN{N}_2\left({G}_2\right)$;
3: Construct ${Z}_{wide}=\left[{Z}_1^{\left({D}_1\right)},{Z}_2^{\left({D}_2\right)}\right]$;
4: Construct neural network (NN) and feed forward, obtain $\hat{y}= NN\left({Z}_{wide}\right)$;
5: Define regularized loss function
$\overset{\sim }{J}\left(W,b\right)= MSE\left(y,\hat{y}\right)+\frac{\lambda }{2}\Big\Vert W{\left.\kern0em \right\Vert}_2^2$ ,
iteratively update $W,b$ as shown in equations (2)–(3);
**end**

To estimate the model parameters, we apply the backward propagation with respect to the mean squared error (MSE) loss function regularized by the ${L}_2$ norm penalty. First, we define the empirical risk function $J\left(W,b\right)$ and the penalty term $\Omega (W)$ as follows. 


$$ J\left(W,b\right)=\frac{1}{n}\sum_{i=1}^nl\left({y}_i,{\hat{y}}_i\right)=\frac{1}{n}\sum_{i=1}^n{\left({y}_i-{\hat{y}}_i\right)}^2,\kern0.5em \Omega (W)=\frac{1}{2}\Vert W{\left.\kern0em \right\Vert}_2^2. $$

The regularized loss function $\overset{\sim }{J}$ is then defined as


(1) \begin{equation*} \overset{\sim }{J}\left(W,b\right)=J\left(W,b\right)+\lambda \Omega \left(W,b\right) \end{equation*}

where $\lambda$ is the penalty parameter determined by the cross-validation technique. The parameters can be estimated by minimizing the regularized loss function with the gradient descent technique. We iteratively update the parameters based on the following equations, until the loss function (1) converges. 


(2) \begin{equation*} W=W-r\frac{\partial \overset{\sim }{J}\left(W,b\right)}{\partial W} \end{equation*}

(3) \begin{equation*} b=b-r\frac{\partial \overset{\sim }{J}\left(W,b\right)}{\partial b} \end{equation*}

Here $r$ represents an adaptive learning rate determined by the ADADELTA algorithm [24]. The technique details can be found in Appendix 2.

Compared to the FLM and FNN models, our model provides a more flexible structure that can easily accommodate multiple omics inputs, consider their nonlinear and nonadditive (e.g. interactions) features, and have advantages of being robust to high dimensionality and high noise levels. The shared representation layer in our model is capable of capturing the correlations between multiple omics inputs and avoids the situation where certain hidden nodes are trained exclusively for one source of omics input. Through the simulation study in section “Results,” we show that these two improvements in our proposed model lead to better prediction performance compared to FLM, NN, or FNN.

## Results

### Simulation studies

Through simulation studies, we evaluate the performance of MFDL for multiomics data analysis and compare it with FLM and FNN. For all simulation studies, to mimic the minor allele frequencies and LD in the real genome, all genotype data was drawn directly from the 1000 Genomes project [25]. We simulated various nonlinear and interactive relationships between the phenotype and omics data to demonstrate the efficiency of MFDL in capturing complex relationships. We also simulated phenotypes in both scalar and vector forms to demonstrate the flexibility of MFDL and introduced various noise levels to show the robustness of MFDL.

#### Simulation settings

For simplicity, we use two types of omics information: genotype data (i.e. SNPs) as ${G}_1\left({t}_1\right)$ and gene expression data as ${G}_2\left({t}_2\right)$, while the method can accommodate various types of omic data. For all the simulations, we used real genetic data from the 1000 Genome project to reflect the real sequencing data structure (e.g. LD pattern and allele frequency). Specifically, we used a 1 Mb region from the genome (Chromosome 17: 7344328–8344327), and randomly chose a 30-kb segment from the 1 Mb region for each simulation replicate to mimic LD patterns and allele frequency distributions from the real genetic data. The minor allele frequency (MAF) of the SNPs in the genome region ranged from $4.50\times{10}^{-4}$ to $4.99\times{10}^{-1}$, with a distribution highly skewed to rare variants (34.8% of the variants with MAF $<.001$, 69.1% of the variants with MAF $<.01$ and 80% of the variants with MAF $<.03$). We randomly select 200 samples ($n=200$) and 100 SNPs (${p}_1$ = 100) from the 30 kb segment to construct ${G}_1\left({t}_1\right)$. Two cases of gene expression data ${G}_2\left({t}_2\right)$ of ${p}_2=1$ and ${p}_2=50$are generated for 200 samples from multivariate normal distributions with $\mu =0,{\sigma}^2=0.5$ for ${p}_2=1$ and $\mu ={\left(0,\dots, 0\right)}_{1\times 50}^T$, $$\Sigma ={\left[\!\begin{array}{ccc}0.5& \cdots & 0\\{}\vdots & \ddots & \vdots \\{}0& \cdots & 0.5\end{array}\!\right]}_{50\times 50}$$ for ${p}_2=50$.

We simulate two types of outcomes: scalar and vector. The relationship between the phenotype outcomes and omics data consists of two functions ${f}_1$ and ${f}_2$ based on ${G}_1$ and ${G}_2$, respectively. Moreover, we consider three types of relationships between omics and outcomes: a linear relationship, a linear relationship with interaction, and a nonlinear relationship.

The linear and nonlinear models are simulated as:


(4) \begin{equation*} {y}_i={y}_{1i}+{y}_{2i}+{\epsilon}_i\kern0.5em ={f}_1\left({G}_{1i}\left({t}_1\right)\right)+{f}_2\left({G}_{2i}\left({t}_2\right)\right)+{\epsilon}_i \end{equation*}

where ${f}_1,{f}_2$ are linear/nonlinear functions when the relationships are linear/nonlinear for a scalar response. For a vector response, the fixed coefficients in ${f}_k\left({G}_{ki}\left({t}_k\right)\right)$ can be simulated in different dimensions to facilitate a vector-to-vector transformation from ${G}_{ki}\left({t}_k\right)$ to ${y}_{ki}$. For the linear relationship, we take


(5) \begin{equation*} {f}_k\left({G}_{ki}\left({t}_k\right)\right)={a}_k{\int}_0^1{G}_{ki}\left({t}_k\right){B}_{k1}\left({t}_k\right){C}_kd{t}_k,\kern0.5em k=1,2. \end{equation*}

For the nonlinear relationship, we define 


(6) \begin{align*} {f}_k\left(s;{G}_{ki}\left({t}_k\right)\right)=\sum\limits_{l=1}^{3}{c}_{kl}{\int}_0^1{G}_{ki}^{e_l}\left({t}_k\right)\mathit{\cos}\left({a}_{kl}{t}_k+{d}_{kl}^{(1)}\right)\nonumber\\\mathit{\sin}\left({a}_{kl}s+{d}_{kl}^{(2)}\right){C}_k\ {dt}_k, \end{align*}

where ${a}_k\sim unif\left(-3,3\right),\left({a}_{k1},{a}_{k2},{a}_{k3}\right)=\left(\frac{2}{3},-2,2\right),\left({c}_{k1},{c}_{k2},{c}_{k2}\right)=\left(\frac{2}{3},-2,2\right),\kern0.5em \left({d}_{kl}^{(1)},{d}_{kl}^{(2)}\right)\sim unif\left(-\pi, \pi \right),$ and $e=\left(\frac{1}{3},\frac{3}{2},3\right)$. ${B}_{k1}\left({t}_k\right)$ is a predetermined fifth-order B-spline basis functions, and ${C}_k$ is a fixed coefficient matrix that takes different dimension according to the data type of ${y}_i$.

To further evaluate interaction effects in the simulation, we introduced an interaction term to the linear transformation, which is defined as the inner product of ${f}_1$ and ${f}_2$ [26],


(7) \begin{align*} In{t}_i= &\ c<{f}_1\left({G}_{1i}\left({t}_1\right)\right),{f}_2\left({G}_{2i}\left({t}_2\right)\right)\gt\nonumber\\=&\ c{\int}_0^1{G}_{1i}\left({t}_1\right){B}_{11}\left({t}_1\right){C}_1d{t}_1{\int}_0^1{G}_{2i}\left({t}_2\right){B}_{21}\left({t}_2\right){C}_2d{t}_2 \end{align*}

where $c$ is chosen as a fixed scalar coefficient. The corresponding linear model with interaction is defined as


(8) \begin{equation*} {y}_i={y}_{1i}+{y}_{2i}+ In{t}_i+{\epsilon}_i, \end{equation*}

where $\epsilon$ is generated from a normal distribution with a mean of 0 and various choices of variance. In all three simulations, we randomly divide the samples into a training set of size 160 and a testing set of size 40. To mitigate the risk of random findings, we replicate each simulation setting 200 times and set a maximum of ${10}^5$ training epochs. To ensure consistency across all models, we use the ${L}_2$ penalty for all models, where the regularization parameter, $\lambda$, is selected from the set $\left\{\mathrm{0.1,0.3,1},3,10\right\}$ using the validation technique.

We compare the performance of MFDL with FLM and deep FNN with three hidden layers (FNN-3HL). Two evaluation criteria are employed: mean square error (MSE) and RV correlation coefficients between the predicted values $\hat{Y}=\left({\hat{y}}_1,\dots, {\hat{y}}_n\right)$ and true values $Y=\left({y}_1,\dots, {y}_n\right)$, defined in the two equations below. The RV correlation coefficient is a multivariate generalization of the squared Pearson correlation coefficient proposed by Robert and Escoufier [27].


$$ MSE\left(Y,\hat{Y}\right)=\frac{1}{n}\sum_{i=1}^n{\left({y}_i-{\hat{y}}_i\right)}^2\ \mathrm{and}\ RV\left(Y,\hat{Y}\right)=\frac{tr\left(Y{Y}^{\prime}\hat{Y}{\hat{Y}}^{\prime}\right)}{\sqrt{tr{\left(Y{Y}^{\prime}\right)}^2 tr{\left(\hat{Y}{\hat{Y}}^{\prime}\right)}^2}}. $$

#### Simulation 1

**Figure 3:**
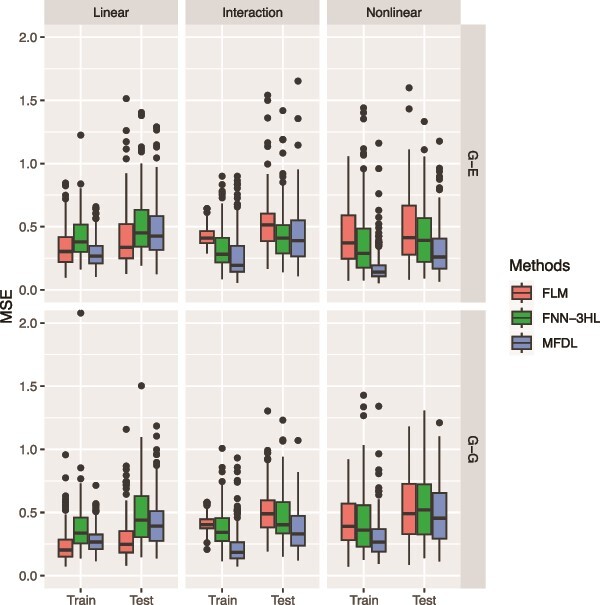
MSE of the three methods under three relationships (the linear, the interaction, and the nonlinear relationships) and two types of omics data (G-E and G-G).

**Figure 4:**
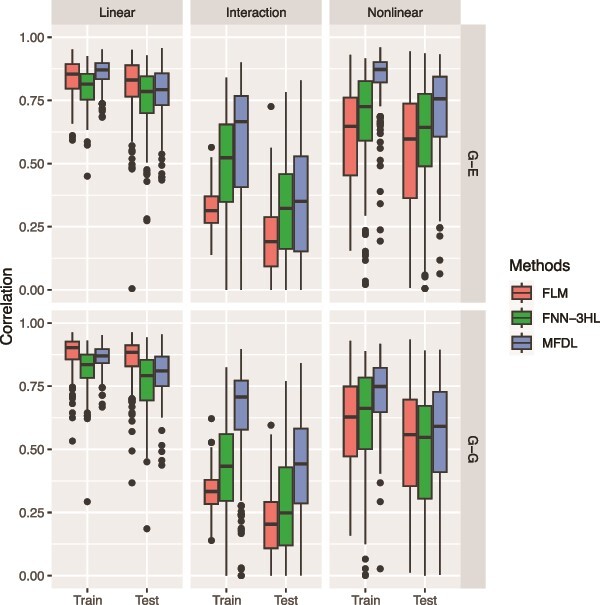
RV correlation coefficients of the three methods under three relationships (the linear, the interaction, and the nonlinear relationships) and two types of omics data (G-E and G-G).

In the first simulation, our aim is to evaluate three types of underlying relationships, a linear, a linear with interaction, and a nonlinear relationship between different omics inputs and a scalar phenotype, with a fixed noise level (i.e. $\mathit{\operatorname{var}}\left({\epsilon}_i\right)=0.3$). We explore two types of omics input data: (i) ${G}_1\left({t}_1\right)$ as vector and ${G}_2\left({t}_2\right)$ as scalar to mimic the Genetic and Gene Expression data (G-E), and (ii) both ${G}_1\left({t}_1\right)$ and ${G}_2\left({t}_2\right)$ as vectors to mimic the Genetic and Genetic data (G-G). The primary distinction between treating omics data as vectors or functional data is whether we account for information (e.g. LD) from neighboring genetic variants. For an omics input treated as a functional input in MFDL, we apply beta-smoothing to the weight parameter in the input layer and vector-to-vector transformation in the hidden layers. We assess the model performances across six scenarios with the two types of omics data and three transformation functions, defined as in (equations (4)–(8)). The results of these six scenarios are shown in Figs 3 and 4.

In Figs 3 and 4, the first row depicts the performance of three methods under various relationships (i.e. a linear, a linear with interaction, and a nonlinear relationship) for the G-E data, while the second row summarizes the results for the G-G data. In the linear setting (left panels of Figs 3 and 4), MFDL and FLM have comparable performance and outperform FNN-3HL for all input data types. When there is an interaction between the omics data (middle panels of Figs 3 and 4), the MFDL model attains higher accuracy than FLM and FNN-3HL in terms of MSE and RV correlation coefficients, particularly in the G-G setting. In cases of nonlinear relationships (right panels of Figs 3 and 4), MFDL again achieves the highest accuracy across all models for both types of omics input data. Overall, the findings suggest that the proposed MFDL model excels at capturing complex nonlinear and nonadditive relationships between outcomes and multiple omics data, while it attains comparable performance to the other two methods in simpler scenarios (e.g. the linear relationship).

#### Simulation 2

In the second simulation, we compare the performance of the three methods across different phenotype types (i.e. scalar and vector) under three types of underlying relationships with the G-E omics data. The noise level is set at 0.3 (i.e. $\mathit{\operatorname{var}}\left({\epsilon}_{\mathrm{i}}\right)=0.3$). For this setup, we generate ${G}_1\left({t}_1\right)$ as functional data with ${p}_1=100$, while ${G}_2\left({t}_2\right)$ is simulated as a scalar. Two types of phenotypes $y$ are simulated: a scalar and a vector of dimension 50.

**Figure 5:**
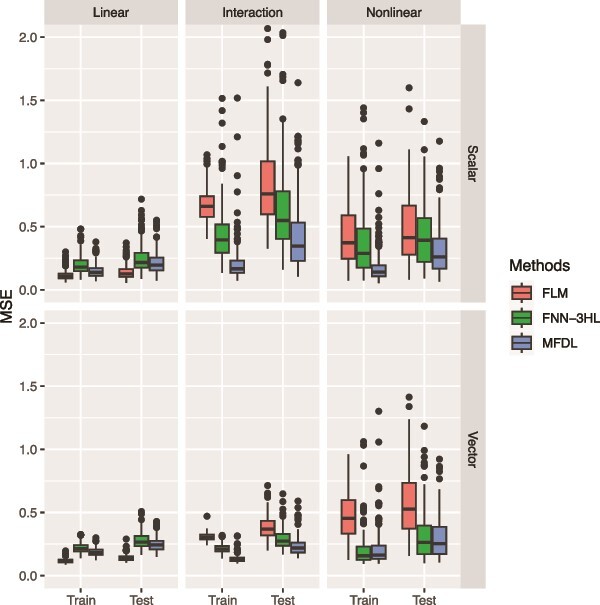
MSE of the three methods under three relationships (the linear, the interaction, and the nonlinear relationships) and two types of phenotypes (scalar and vector phenotypes).

**Figure 6:**
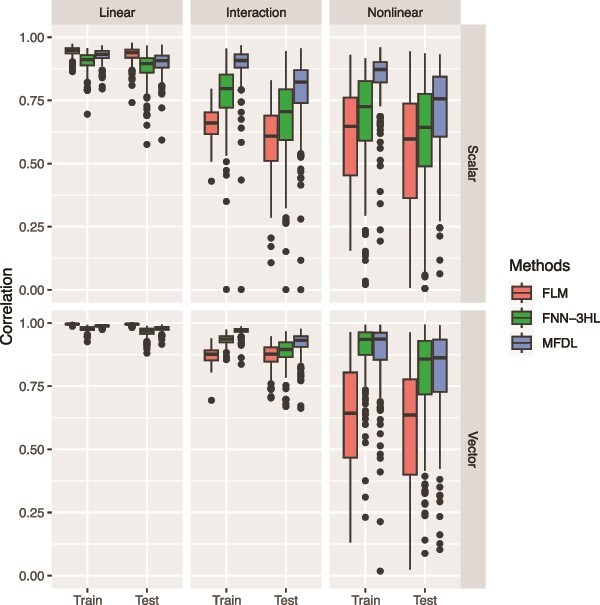
RV correlation coefficients of the three methods under three relationships (the linear, the interaction, and the nonlinear relationships) and two types of phenotypes (scalar and vector phenotypes).

Similar to the results in simulation 1, MFDL attains better or at least comparable performance than FLM and FNN-3HL across different phenotype types, underlying relationships, and omics data. Additionally, both MFDL and FLM outperform FNN-3HL under the linear relationship (left panels of Figs 5 and 6), while both MFDL and FNN-3HL outperform FLM with the vector phenotypes and nonlinear relationship (right bottom panel of Figs 5 and 6).

#### Simulation 3

**Figure 7:**
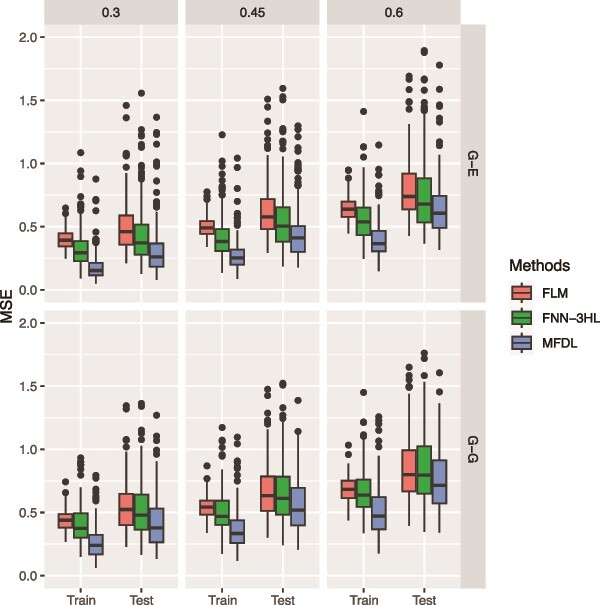
MSE of the three methods under three noise levels (0.3, 0.45, and 0.6) and two types of omics data (G-E and G-G).

**Figure 8:**
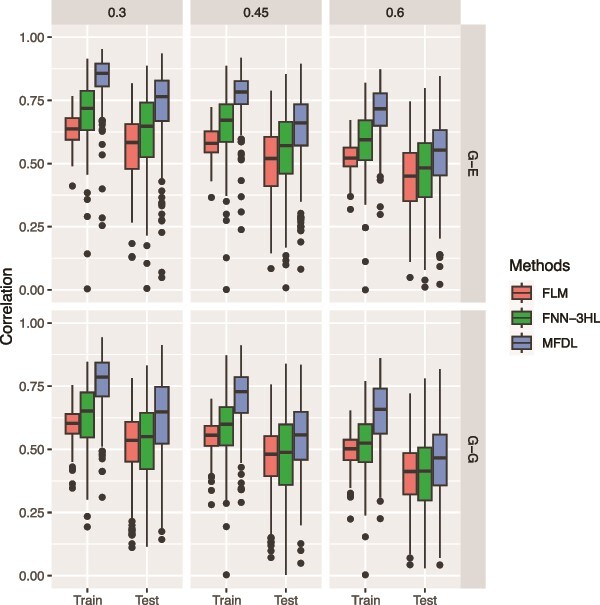
RV correlation of the three methods under three noise levels (0.3, 0.45, and 0.6) and two types of omics data (G-E and G-G).

In the third simulation, we evaluate the robustness of the three methods with increasing levels of noise, mimicking the high noise-signal ratio in real-world multiomics data. Specifically, we simulate three noise levels: 0.3, 0.45, and 0.6. (i.e. $\mathit{\operatorname{var}}\left({\epsilon}_{\mathrm{i}}\right)=\left\{\mathrm{0.3,0.45,0.6}\right\}$). In this simulation, we considered scalar phenotypes, the linear relationship with interactions, and both G-G and G-E omics data settings. The omics input data are generated as described in simulation 1. Figures 7 and 8 show that the proposed MFDL model achieves the smallest MSE and the highest RV correlation in all six scenarios, indicating the robustness of the MFDL model against various noise levels.

In conclusion, through three simulations, we demonstrate the MFDL model’s ability to capture complex relationships between different types of phenotypes and multiomics data. With the advantage of the multimodal structure, MFDL provides a more effective way of capturing the latent features from various omics data and modeling the interaction effect between multiple omics. Additionally, our proposed model demonstrates robustness against various noise levels and high-dimensional omics and phenotype data.

### Real data application

Alzheimer's disease (AD) is a progressive neurodegenerative disorder characterized by its complex and multifactorial nature. Although numerous studies have explored the role of various omics data in AD, the combined effects of multilevel omics data remain underevaluated. In this study, we undertake an integrative analysis of DNA sequencing and gene expression data derived from the AD Neuroimaging Initiative (ADNI) project. ADNI is a multisite study designed to evaluate clinical, imaging, genetic, and biospecimen biomarkers across the spectrum of normal aging to early mild cognitive impairment (MCI) and AD. DNA samples from 808 participants were subjected to non-CLIA whole-genome sequencing (WGS) at Illumina.

For our phenotype of interest, we focus on hippocampal volume changes observed in structural MRI scans, a critical marker for Late-Onset AD (LOAD), examining the contributions of genetic, gene expression, and biomarker variations to these changes over time.

Before analysis, per-individual quality control (QC) and per-marker QC [28] were implemented. The per-individual QC excludes samples with massive missing genotype or related to other individuals. The per-maker QC excludes SNPs with insufficient proportion of successful genotype calls, marks that shows significant deviation from Hardy–Weinberg equilibrium (HWE) or with a very low minor allele frequency.

#### Predicting hippocampus volume change over time with Apolipoprotein E (APOE) genotype, gene expression, and biomarker data

To prepare the multiomics dataset for the analysis, we select three omics inputs: *APOE* genotypes, *APOE* gene expression levels and biomarker “A$\beta$-42” [29], which are recognized to affect AD pathology. We extracted the *APOE* genotypes, corresponding gene expression levels and biomarker A$\beta$-42 for all 808 participants from the ADNI dataset. This omics data were then integrated with longitudinal measurements of hippocampal volume derived from the ADNI structural MRI data. Participants who had only a single hippocampal volume measurement were excluded, resulting in a final dataset comprising 370 individuals and 1456 hippocampal volume measurements, along with the participants’ ages at each visit.

We applied four methods to the omics dataset from the ADNI, including FLM, DL model, FNN-3HL, and the proposed MFDL. These methods were used to investigate the combined effects of *APOE* genotypes, gene expression and the biomarker A$\beta$-42 on the hippocampus volume change over time. In FLM, the omics inputs are modeled as three separate terms, as detailed in section “Functional Linear Model.” The DL model treats the three data matrices in vector form and concatenates them column-wise prior to training the neural network. For the FNN-3HL model, genotype data are modeled in a functional form, and the discretization of the genetic variant function is then combined with the gene expression data and biomarker data for model fitting. For MFDL, the three omics inputs are trained independently to construct the shared information layer.

The phenotype comprises two or more observations of hippocampus volume change per participant taken during their visits and is insufficient to construct a function across these points. Consequently, the phenotype is treated in vector form, and the patients’ age at the time of their first visit is used as a covariate in all models. Similar to the simulation studies, 278 patients were randomly selected to train the models, while the remaining 92 patients were used as the test set. The models are evaluated and compared using the MSE, MAE, and RV correlation coefficients between the observed and predicted phenotypes. To mitigate the effects of random data splitting, we repeated the process 200 times for each model with three-fold crossvalidation on the training set.

**Figure 9:**
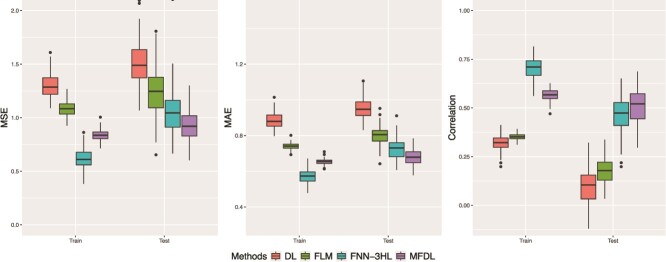
Prediction of the change of hippocampus volume using APOE genotypes, gene expression, and the biomarker Aβ-42.

Figure 9 shows that the MFDL model achieves superior performance compared to other methods on the test set regarding all the three criteria (MSE, MAE, and RV correlation). Moreover, compared to deep FNN models, our proposed MFDL model exhibits considerably less overfitting by comparing the performance between the training and test sets.

#### The effect of *APOE–ACE* interaction on predicting hippocampus volume change over time

In this part of the study, we study a gene–gene interaction related to hippocampus volume change over time. We consider *ACE*, which has been previously identified as having a strong association with LOAD and exhibits gene–gene interactions with the *APOE4* allele status [30]. By applying our methods to the ADNI dataset, we aim to evaluate the impact of the interaction between *APOE* and *ACE* on the prediction of hippocampal volume changes over time. *ACE* genotypes were sourced from the ADNI dataset. Following the same data processing as in “Predicting hippocampus volume change over time with Apolipoprotein E (APOE) genotype, gene expression and biomarker data,” a total of 625 samples with 1250 hippocampus volume measurements were retained for analysis. We extracted SNPs from the *APOE* and *ACE* genotypes, along with their SNP location information, from the ADNI dataset, which were modeled as functional inputs in the FLM, FNN, and MFDL. Similar to the analysis in section “Predicting hippocampus volume change over time with Apolipoprotein E (APOE) genotype, gene expression and biomarker data,” we repeated the modeling process 200 times for each model with three-fold crossvalidation on the training set.

**Figure 10:**
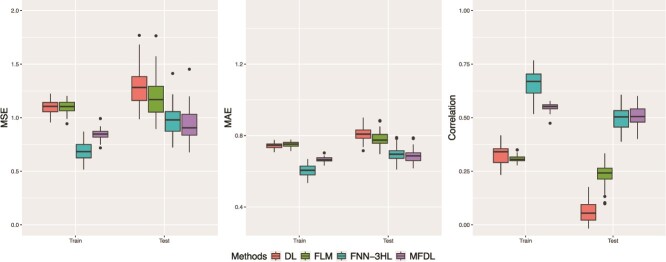
Prediction of the change of hippocampus volume by considering an interaction between APOE and ACE.

Figure 10 shows that our proposed method surpasses the existing FLM, DL, and FNN in terms of testing MSE, MAE, and RV correlation coefficient performance. Additionally, the difference of the training and testing results may suggest that DL and FNN-3HL are susceptible to overfitting. In contrast, our proposed model exhibits robust performance. Compared to data from section “Predicting hippocampus volume change over time with Apolipoprotein E (APOE) genotype, gene expression and biomarker data,” there is an increase of data dimensionality in the two genotypes and possibly the noise level, the MFDL still consistently captures the gene–gene interaction and is less prone to the overfitting issue.

## Discussion

In this paper, we introduce a novel multimodal functional DL method for the analysis of high-dimensional multiomics data. The proposed MFDL method uses the hierarchy of neural networks to learn complicated features from omic data, making it more powerful to model complex relationships (e.g. interactions between omics) than the tradition methods, such as FLM. By modeling effects as a function in the form of a combination of basis functions, MFDL is able to take information from nearby markers into account and reduce model complexity, providing more robust performance than DL for high-dimensional omic data analysis. By using a shared representation layer, the MFDL model is flexible to handle different types of omic data. Unlike existing methods, such as FNN, MFDL uses subnets to model each omic data and model their complex relationships based on the shared representation layer. Such a strategy not only provides flexibility to accommodate different data types (e.g. functional data versus nonfunctional data) but also reduces the complexity of the network structure.

Through simulation studies and real-world data applications, the proposed model has demonstrated superiority over both the FLM and FNN models in scenarios where multiomics data exhibit complex relationships (e.g. nonlinear relationships and interactions). The MFDL model also exhibits robustness in scenarios with increasing noise levels or high-dimensional data. In comparison with the FNN model, which is prone to overfitting under certain conditions, our proposed MFDL model is more adept at handling multiomics data.

In a traditional feedforward neural network with fully connected layers, when multiple inputs are merged to train the model, the hidden nodes in the network tend to become exclusively attuned to one type of the input. For example, in the case of a two-omics-input scenario, after certain training iterations, some hidden nodes may predominantly relate to the first type of input, while others are more closely associated with the second type of input. This tendency presents challenges for the neural network in capturing strong interactions between omics data, which can result in poor performance. Although FNN leverages the smoothness of genetic information to enhance predictive performance, it still struggles to identify a functional transformation that encapsulates the internal relationships among multiple types of inputs. The multimodal structure with its shared representative layer offers an effective solution for modalities that have latent interactions as demonstrated in multimodal DL methods applied to video–audio datasets [31]. The adaptability of its structure, combined with the robustness of the shared representative layer, positions our proposed model as a useful tool for modeling multiomics data.

MFDL can also be further extended to consider functional phenotypes (e.g. imaging and time-dependent phenotypes). For a single genetic input, FNN addresses this issue by converting vector-to-vector transformations between hidden layers into function-to-function transformations. However, for multiple omics inputs, the main challenge faced by FNN is fitting a function on both functional (e.g. SNPs) and nonfunctional data (e.g. gene expression), in which almost no basis systems are suitable for both data. Moreover, FNN becomes problematic in defining a function from the shared representative since the location information is not unified across different omics data. Consequently, our proposed model faces the same challenges when dealing with functional phenotypes. While a simple solution is treating a functional phenotype as a vector, exploring alternative strategies that incorporate additional information (e.g. location and temporal information, and networks) is worthwhile for future investigation. Statistical testing building on MFDL holds great promise for rigorously evaluating the complex associations between multiomics with the phenotype of interest and result interpretation. This represents an important avenue for further research.

**Key Points**

- We develop an MFDL approach to model the complex relationships between multiomics and disease phenotypes.
- The MFDL approach imposes a hierarchical structure of deep neural networks using the individually trained functional neural network on each omics feature to build a shared representation layer that encapsulates complex interactions between omics features.
- The new approach shows superior prediction performance for complex nonlinear relationships and interactions among omics data, as well as robustness to the high dimensionality and noise levels within the data.

## Supplementary Material

MFDL_Appendix_bbae448

## Data Availability

The source code for the simulation and real data application is available at https://github.com/DianaYuanZhou/MFDL.git. The ADNI dataset used in this article is available at adni.loni.usc.edu.
