# Supplementary material for: Multimodal functional deep learning for multiomics data
Source: Brief Bioinform. 2024 Sep 16;25(5):bbae448. doi: 10.1093/bib/bbae448 (PMC11405129; doi:10.1093/bib/bbae448)
Supplement: MFDL_Appendix_bbae448 [file mfdl_appendix_bbae448.pdf]

Appendix A. Forward Propagation of Multimodal Functional Deep Learning

Step 1: Separate Training

The explicit form of the bias and weight function of this step can be expressed as

$$\beta_k^{(d_k)}\left(t_k^{(d_k)}, t_k^{(d_k-1)}\right) = \sum_{j=1}^{J^{(d_k)}} \sum_{\ell=1}^{J^{(d_k-1)}} w_{\ell j}^{(d_k)} \eta_\ell^{(d_k-1)}(t_k^{(d_k-1)}) \eta_j^{(d_k)}(t_k^{(d_k)})$$

$$\alpha_{k0}^{(d_k)}\left(t_k^{(d_k)}\right) = \sum_{j=1}^{J^{(d_k)}} b_j^{(d_k)} \eta_j^{(d_k)}(t_k^{(d_k)}).$$

Rewrite the functional notation in matrix form as follows:

$$B_k^{(d_k)} = \left[\eta_j^{(d_k)}(t_{km}^{(d_k)})\right]_{m=1,j=1}^{p^{(d_k)},J^{(d_k)}}$$

$$Z_k^{(d_k)} = \left[Z_{ki}^{(d_k)}(t_{km}^{(d_k)})\right]_{i=1,m=1}^{n,p^{(d_k)}}.$$

For input matrix $G_k(t_k), k = 1,2$, the forward propagation can be rewrite as

$$Z_k^{(0)} = G_k$$

$$D_k^{(d)} = Z_k^{(d_k-1)} B_k^{(d_k-1)} / p^{(d_k-1)}$$

$$A_k^{(1)} = D_k^{(1)} w_k^{(1)} + 1_{n,1} b_k^{(1)}$$

$$A_k^{(d_k)} = D_k^{(d_k)} w_k^{(d_k)} + 1_{n,1} b_k^{(d_k)}, d_k > 1$$

$$Z_k^{(d_k)} = \sigma\left(A_k^{(d_k)} B_i^{(d_k)}\right).$$

For a non-functional input, we apply the basis B_k as diagonal matrix. In this case, the FNN is reduced to a Deep Neural Network model.

Step 2: Combined Training

The output $Z_1^{(D1)}, Z_2^{(D_2)}$ from the first step are concatenated as

$$Z^{(1)} = Z_{wide} = \left[Z_1^{(D1)} \quad Z_2^{(D_2)}\right].$$

Then the forward propagation of Deep Neural Network in this step can be expressed as follows

$$A^{(d)} = Z^{(d-1)} w^{(d)} + 1_{n,1} b^{(d)}, d > 1$$

$$Z^{(d)} = \sigma\left(A^{(d)}\right)$$

$$\hat{Y} = Z^{(D)}.$$

Appendix B. Backward Propagation of Multimodal Functional Deep Learning

As defined in function (2.2), $\tilde{J}(W,b) = J(W,b) + \lambda\Omega(W,b)$, where

$$J(W,b) = \frac{1}{n}\sum_{i=1}^{n} l(y_i,\hat{y}_i) = \frac{1}{n}\sum_{i=1}^{n}(y_i - \hat{y}_i)^2 , \Omega(\mathrm{W}) = \frac{1}{2}\|W\|_2^2.$$

We start with the partial derivative of $J(W,b)$. We define $\mathrm{M}^{(\mathrm{d})} = \frac{\partial J(W,b)}{\partial \mathrm{A}^{(d)}}$ and calculate the partial derivative terms in two steps.

Step 1: Calculate the partial derivative of the 'combined training' step.

$$M^{(D)} = 2(\hat{Y} - Y)$$

$$M^{(d)} = \left(M^{(d+1)}w^{(d+1)T}\right) \circ \sigma'\left(A^{(d)}\right), d < D$$

$$\frac{\partial J}{\partial w^{(d)}} = \left(Z^{(d_2-1)}\right)^T M^{(d)}$$

$$\frac{\partial J}{\partial w^{(1)}} = (Z_{wide})^T M^{(1)}$$

$$\frac{\partial J}{\partial b^{(d)}} = 1_{1,n} M^{(d)}.$$

Step 2: Calculate partial derivatives for each FNN model in 'combined training' step separately. To begin with, we rewrite $\mathrm{Z_{wide}}$ with the following equation:

$$Z_{wide} = \sigma\left(\left[A_1^{(D_1)}B_1^{(D_1)}, A_2^{(D_1)}B_2^{(D_1)}\right]\right)$$

$$= Z_1^{(D_1)}I_1 + Z_2^{(D_1)}I_2,$$

where $I_1 = \left[I_{p_{d_1}*p_{d_1}} \quad 0_{p_{d_1}*p_{d_2}}\right]$ and $I_2 = \left[0_{p_{d_2}*p_{d_1}} \quad I_{p_{d_2}*p_{d_2}}\right]$. p_{d_1} and p_{d_2} are the dimensions of $\mathrm{Z}_1^{(\mathrm{D}_1)}$ and $\mathrm{Z}_2^{(\mathrm{D}_1)}$. Then partial derivative can be expressed as

$$M_k^{(D_k)} = \left(M_k^{(1)}w^{(1)T}B^{(1)T}I_i^T\right) \circ \sigma'\ \left(A_k^{(D_k)}B_k^{(D_k)T}\right)B^{(D_k)}$$

$$M_k^{(d_k)} = \left(M_k^{(d_k+1)}w^{(d_k+1)T}B^{(d_k)T}\right) \circ \sigma'\ \left(A^{(d_k)}B^{(d_k)T}\right)B^{(d_k)}$$

$$\frac{\partial J}{\partial w_k^{(d_k)}} = \frac{\left(Z_k^{(d_k-1)}B_k^{(d_k-1)}\right)^T M_k^{(d_k)}}{p^{(d_k-1)}} + (d_k - 1), d_k > 1$$

$$\frac{\partial J}{\partial w_k^{(1)}} = \left(Z_k^{(1)}B_k^{(1)}\right)^T M_i^{(1)}/p_k^{(0)}$$

$$\frac{\partial J}{\partial b_k^{(d_k)}} = 1_{1,n_i}M_k^{(d_k)} \text{ (2.33).}$$

It is straight forwarded to derive

$$\frac{\partial J}{\partial w_k^{(d_k)}} = \lambda w_k^{(d_k)} \text{ (2.34)}$$

$$\frac{\partial J}{\partial w^{(d)}} = \lambda w^{(d)} \text{ (2.35)}$$

Then, the update process of the parameters can be rewritten as

$$W = W - \alpha\left(\frac{\partial J(W,b)}{\partial W} + \lambda W\right) \text{ (2.36)}$$

$$b = b - \alpha\frac{\partial J(W,b)}{\partial b} \text{ (2.37)}$$
